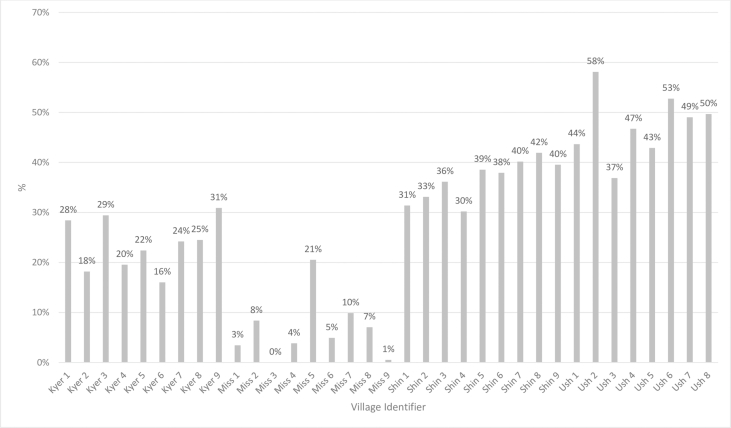# Community Health Workers’ Targeting of Women For Health and Nutrition Home Visits in Rural Tanzania: A Mixed-Methods Study

**DOI:** 10.1016/j.cdnut.2024.103780

**Published:** 2024-05-18

**Authors:** Ibukun Owoputi, John Hoddinott, Rosemary Kayanda, Rachel Bezner Kerr, Kate Dickin

**Affiliations:** 1 Division of Nutritional Sciences, Cornell University, Ithaca, NY, United States; 2 Department of Global Development, Cornell University, Ithaca, NY, United States; 3 Charles H. Dyson School of Applied Economics and Management, Cornell University, Ithaca, NY, United States; 4 IMA World Health, Mwanza, Tanzania; 5 Department of Public and Ecosystem Health, Cornell University, Ithaca, NY, United States

**Keywords:** implementation research, community health workers, home visits, maternal and child nutrition, GIS, GPS, Tanzania

## Abstract

**Background:**

Community health workers (CHWs) are utilized in many health systems to provide education and messaging to families in their catchment areas. However, CHWs responsible for large geographic areas often must make important decisions about whom to visit. Factors that influence these decisions are understudied.

**Objectives:**

This study assessed coverage and targeting for home visits by CHWs within a large social and behavioral change health program in rural Tanzania.

**Methods:**

This implementation research was a cross-sectional, mixed-methods study. Data collection included a census with households and surveys with females, surveys with CHWs, and interviews with CHWs. Survey data also included the collection of household location data for females and CHWs. Quantitative data were analyzed using linear probability models, and qualitative data were analyzed using inductive thematic analysis.

**Results:**

Only 13% of eligible households in our study sites reported receiving a home visit from a CHW. Although CHWs were more likely to reach households with infants, other program priority populations, such as poor and food insecure households, were frequently missed. Global positioning system data showed that distance was 1 of the greatest barriers for CHWs in providing home visits. Qualitative data indicated that although CHWs were motivated and engaged to improve maternal and child health in their communities, they faced challenges in visiting households that were further away or lacked economic resources to improve their health behaviors. CHWs also found it difficult to provide health education during home visits to mothers with no formal schooling.

**Conclusions:**

Programs relying on community volunteers need to set realistic workloads, especially when volunteer CHWs also work full-time in their primary occupations. Implementation could also be strengthened by providing extra support for CHWs so that they can effectively provide services to community members who are more difficult to visit but may be the most in need.

## Introduction

Although Tanzania has drastically reduced infant mortality (from 143 to 35/1000 births between 1960 and 2020 [1], malnutrition continues to be a serious concern. As of 2015/2016, childhood stunting (low height-for-age) in Tanzania remained at 34% [2,3]. The factors contributing to stunting are multisectoral and complex, including maternal malnutrition, poor infant feeding practices, poor health care, and poor hygiene and sanitation practices [4]. In Tanzania, sub-optimal feeding behaviors are ubiquitous. As of 2018, only 35% of children under the age of 2 had an adequate minimum dietary diversity, and only 30% received a minimum acceptable diet [5]. In another study, researchers found that exclusive breastfeeding was extremely rare, as most females reported giving prelacteals or sugar water in the first few days after birth [6]. In addition, de Bruyn et al. [7] found that child intake closely followed the mother’s intake in terms of poor dietary diversity in Tanzania. Maternal short stature and low BMI (in kg/m^2^) are associated with fetal growth restriction, indicating the cycle of malnutrition that occurs in the Global South [8]. Many females in Tanzania suffer from micronutrient deficiencies, including vitamin A, iron, and iodine [2].

Many social and behavioral change (SBC) interventions have been designed to address the issue of poor maternal and child nutrition in the Global South [9]. SBC is designed to improve change behaviors by addressing knowledge, attitudes, and norms, as increasing knowledge alone does not lead to sustained behavioral change [9]. SBC has been found to improve females’ dietary practices during pregnancy and lactation, from breastfeeding practices to complementary feeding practices-which are all behaviors associated with stunting in early life [9].

One way that SBC interventions have been designed to improve maternal and child nutrition is through the use of community health workers (CHWs), who provide education, motivate, and problem-solve with clients during home visits. Although they receive less training than formal health care workers, they are supported by their health system and, ideally, are selected by their own communities [10,11] and, thus, are seen to be accountable to their community. CHWs are intended to bridge the gap between the health care system and communities [12]. These workers can also be agents of social change as was the case in Tanzania in the early postcolonial period [13]. Subsequently, however, their role has become more technical [10] and has been used to fill the gap left by the inadequate number of health professionals in Tanzania [14]. A study by Lema et al. [15] found that CHWs in Tanzania can be effective in identifying pregnant females before they attend health care visits, as well as improving breastfeeding rates through breastfeeding promotion [15]. Another systematic review of randomized control trials by Lewin et al. [16,17] showed that breastfeeding promotion by lay health care workers significantly improved exclusive breastfeeding rates in the Global South. In addition, during the COVID-19 pandemic, CHW roles were often expanded to help the over-burdened health care system [[18], [19], [20]].

Although the role of CHWs is well understood and there is some evidence of effectiveness, much less is known about which groups interact with and benefit from CHWs. Factors such as religion, proximity of CHW to household, and socioeconomic status of both the CHW and the household may all play a role [21,22]. Within provider-client interactions such as home visits, providers tend to prefer to interact with people with similar demographic characteristics to themselves, a phenomenon known as homophily [23,24]. It is not known whether homophily applies to CHW programs and influences CHW activities, although 1 study suggests that CHWs from poorer households may be more likely to visit other poor households [21]. Additionally, sociodemographic characteristics can impact the quality of home visits provided by CHWs [25]. A cross-sectional study in Kenya found that the age of the CHW was positively associated with client enablement (the ability of the consultation to result in behavioral change, assessed during exit interviews). In addition, female CHWs were more likely to counsel and enable their clients [25]. In general, many CHW programs consist of CHWs who are older, married females [10].

We assess how CHWs target mothers and households, drawing on data collected in the context of a large maternal and child nutrition program in rural Tanzania, Addressing Stunting Early in Tanzania (ASTUTE). ASTUTE CHWs were required to visit households and deliver relevant messages on several different topics (e.g., breastfeeding practices, dietary diversity, water, sanitation, and hygiene, females’ workload, and early childhood development behaviors). They were given a relatively large amount of autonomy in determining which households to visit and how many times to visit each household. The CHWs were given a small stipend by the program to conduct these home visits. We seek to answer 3 questions: *1*) What is the coverage of CHWs within the ASTUTE program? *2*) Do the CHWs visit those who are the most in need, and how do they determine who to visit/target? and *3*) Do the CHWs visit those who are similar to them demographically (i.e., homophily)?

## Methods

### ASTUTE project overview

The ASTUTE program, designed and implemented by IMA World Health, aimed to reduce the number of stunted children in 5 regions of Tanzania. ASTUTE targeted 1000-d households, the time period from conception to age 2 y, where mothers and children are the most vulnerable [26]. Programming included several types of interventions, including home visits, training and support for health facility workers, and mass media. ASTUTE relied on CHWs to give nutrition and health education during home visits.

### Background on ASTUTE project CHWs

Most of the ASTUTE CHWs implementing the home visits had already been operating as CHWs for various types of programs in the past. The CHWs were managed by CHW supervisors, a new position created by ASTUTE, to identify households for home visits. CHW supervisors’ roles were strictly to supervise CHWs, and they did not implement any home visits themselves. ASTUTE distributed job advertisements for prospective CHWs and CHW supervisors, and the finalists were elected by the local government leaders. For ASTUTE, every village was supposed to have 2 CHWs who had received training. However, some villages had 1 or no trained CHW due to absence during training or a recent replacement for a CHW who had quit. Trained CHWs and CHW supervisors had received 3 d of training. The CHWs were trained in using GALIDRAA (greet, ask, listen, discuss, recommend, agree, and appoint), a communication method designed to help improve behaviors in health SBC communication programs [27]. CHWs were also trained to identify acute malnutrition cases and refer them to the health facilities.

Prior to beginning home visits, the CHWs were responsible for registering all households in their villages (anywhere from 50–360 households) to find eligible pregnant females and mothers of children under the age of 2. CHWs were also responsible for finding and registering newly pregnant females. CHWs were required to complete 6 home visits a week or 24 home visits a month. Half of the 6 weekly home visits were supposed to be new households, and the other half were supposed to be follow up visits.

Due to the large number of eligible households in the registries, the program provided guidelines on who the CHWs should prioritize for home visits: *1*) households where a preschool child was or had been mildly or moderately undernourished; *2*) households participating in Tanzanian Social Action Fund (TASAF) (a government unconditional cash transfer program given to poor or vulnerable households, households with children or pregnant females, and households with disabled family members); *3*) mothers in their first pregnancy; and *4*) households with children aged 3–9 mo.

ASTUTE operated under the model of “supportive supervision,” where the CHW supervisors accompanied each of their CHWs for a home visit once a month. Each CHW supervisor was responsible for anywhere from 7 to 20 CHWs, depending on the number of villages in their ward (Tanzania administrative subdivision). The CHWs were supposed to negotiate with each household the number of home visits they would receive, and then follow up with their CHW supervisor during their monthly visit. During these monthly visits, the CHW supervisors were to ensure that the CHWs were visiting females who met the priority guidelines, collect the CHWs’ visit records, and ensure that CHWs were targeting participants according to priority guidelines and meeting the required number of visits per month.

Prior to beginning this research project, we collected data from the latest ASTUTE quarterly report on the number of households that the CHWs reported visiting their CHW supervisor in each of our study districts (Table 1).

**TABLE 1:** Community health worker home visits from the July-September 2018 quarterly report

Region	District	# of total quarterly visits, per CHW
Kagera	Missenyi	26
	Kyerwa	22
Shinyanga	Ushetu	23
	Shinyanga DC	24
Average		24

Abbreviation: CHW, Community health worker; DC, district council.

CHWs were supposed to perform 6 visits a week (or ∼1/d); in a 3-mo period, they would have been expected to perform (6 x 12 = 72) visits per quarter. Table 1 shows that the actual mean number of visits were only one-third of this target.

CHWs were paid TZS (Tanzanian Shillings) 15,000/mo for their work [which was the equivalent of ∼$6.50 USD (United States Dollars)]. Due to programming delays CHWs often went months without any payment. For comparison, the CHW supervisors were paid TZS 100,000/mo (∼$43 USD).

### Research study setting

The implementation research project presented in this article was conducted by external researchers to the ASTUTE program and took place from October 2018 to February 2019, ∼3 y after the home visits intervention with CHWs had begun.

### Site selection

In Tanzania, administrative subdivisions work as follows: a group of villages make up a ward, a collection of wards make up a district, and a group of districts make up a region. Detailed site selection is below in Table 2.

**TABLE 2:** Site selection and sampling

Administrative subdivision	Number in sample	Name	Sampling approach and criteria
Region	2	Kagera Shinyanga	- Chosen from the 5 ASTUTE implementation regions, with input from the government - Criteria: strong implementation (highest levels of average home visits/CHWs) from the annual report - Chosen to include regions that use the predominant staple crops in Tanzania (1 maize crop region and 1 banana region)
District	4 (2 per region)	Shinyanga DC Ushetu Missenyi Kyerwa	- Purposeful selection, with input from government officials - Rural districts only - CHWs were trained ≥6 mo ago - Districts were selected from both the first year (2) and second year of implementation of the ASTUTE program (2) - Aimed for districts with the highest number of home visits per CHW per quarter
Wards	12 (3 per district)	[redacted]	- Rural wards only, randomly sampled - 2 excluded due to being a part of our previous research studies - 2 wards were excluded due to rainfall/travel safety
Villages	35 (2–3 per ward)	[redacted]	- Chosen randomly

Abbreviations: ASTUTE, addressing stunting early in Tanzania; CHW, community health worker.

### Research activities

All data collection was conducted in the local language (Swahili). The qualitative and quantitative data in this research project were concomitant and were collected at the same time. The qualitative analysis was completed before the quantitative analysis, but the results from each method were integrated for both analysis and interpretation [28].

### Quantitative data collection

#### Census with households and surveys with females

We conducted a household census gathering information on the number of pregnant females or mothers/female caretakers of children under the age of 30 mo (the study pertained to home visits in the past 6 mo, and the program targeted children under 24 mo) currently living in the household. Data collectors worked with local leaders to facilitate conducting the house-to-house census and, to avoid bias, did not involve CHWs except in 2 instances when local leaders were unable or unwilling to assist.

**FIGURE 1:**
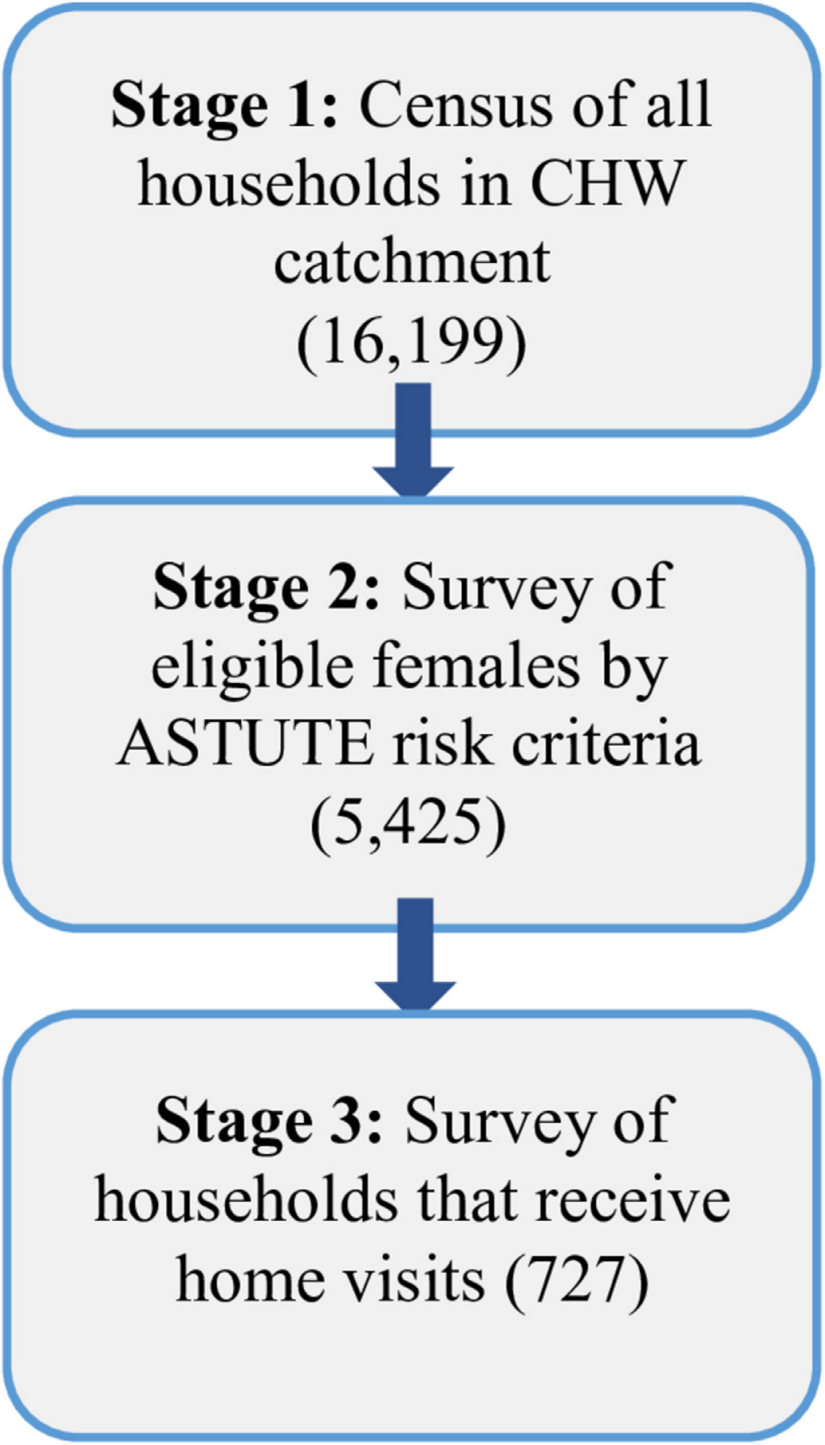
Census/survey staging. ASTUTE, addressing stunting early in Tanzania; CHW, community health worker.

The census/survey was conducted in 3 stages (Figure 1). Stage 1 of the census questionnaire assessed whether the household was eligible for an ASTUTE home visit based on the presence of a pregnant female or mother/female caretaker aged 15 or older who was responsible for a child under 2 y of age. We included female caretakers in addition to mothers because the CHWs were responsible for visiting grandmothers or other female family members who were caring for young children. Households that met the criteria moved on to the survey (stage 2). In stage 2, we collected household information (e.g., household size, number of children under 18), mother-level data (e.g., age, education), and child-level data (e.g., age of child). Survey questions on demographic characteristics (such as household size, education status, and asset ownership) were taken from the Tanzanian demographic and health surveys [2]. For household-level variables such as wealth and food security, these data were only collected from the first available eligible female in a household. We collected global positioning system (GPS) data at the end of the census for households that met the census criteria to identify if the proximity of CHW affects the likelihood of receiving a home visit from a CHW. We also identified females who had received a home visit by asking, “Have you received a home visit in the last 6 mo by a CHW? By CHW, I mean someone who comes to people’s homes and talks about the child’s health, including nutrition?” Households that reported receiving a home visit from a CHW in the past 6 mo were included in stage 3 of the survey which asked additional questions about their experience with the home visits and the name of the CHW that visited them.

The enumerator asked the census questions from any person in the household but asked the female of the household to respond to the survey questions in stage 2 or stage 3. The enumerator asked to speak to all females in a household meeting the census criteria. Research staff collected this survey data and GPS data using tablets and electronic data collection software Open Data Kit (ODK). ODK is an open-source Android-based web application used to design and implement questionnaires.

#### Surveys with CHWs

All CHWs in our study areas were approached at their homes to complete the survey. Although there were supposed to be 2 CHWs per village, in some villages, there was only 1 CHW because the other 1 had quit. Therefore, we surveyed a total of 66 CHWs. The CHW survey collected data on the CHW’s demographic and household information, their experience with implementing home visits, and GPS coordinate data of the CHWs’ homes. Research staff also collected this survey data and GPS data using tablets and ODK.

### Qualitative data collection

#### In-depth interviews with CHWs and CHW supervisors

We purposefully selected a subset of 20 CHWs to interview, either at home or the village office, based on their age, sex, length of time working as a CHW, and village size. We also sampled 6 CHW supervisors (3 males and 3 females) for in-depth interviews. The total sample sizes for the CHWs and CHW supervisor interviews were determined by theoretical saturation [29]. After interviewing the 26 CHWs and CHW supervisors, the preliminary analysis indicated that additional interviews would be unlikely to lead to more insights or new perspectives from our interviewees. We interviewed CHWs and CHW supervisors using a translated and pretested in-depth interview guide exploring how they conducted home visits and why, as well as how they felt about their work. The interview questions were open-ended to allow respondents’ own views to emerge.

### Data analysis

#### Quantitative analysis

Quantitative data were analyzed using STATA SE (v15.1). GPS data were analyzed using QGIS (v3.24.1), which supports the analysis of geospatial data. The datasets were cleaned, and open-ended responses were translated from Swahili into English. The descriptive data were analyzed for females (mothers and female caretakers) and CHWs across relevant variables. Bivariate analysis was completed using χ^2^ for binary variables and Mann-Whitney tests for ordinal variables. The outcome variable was a binary variable: whether or not a female reported receiving a home visit (and remembered the name of their CHW). All regression models controlled for village-level clustering, to account for differences in performance of CHWs, who varied by village. The categories of covariates included household-level, mother-level, child, the distance between GPS locations of CHWs’ and females’ homes, and homophily (e.g., the difference between CHW and females’ age, education, wealth quintile, and ethnic group) data.

#### Bivariate and multiple variable analysis

We completed bivariate analysis to compare household and demographic data with the likelihood of a female being visited by a CHW to determine which variables to keep in the linear probability models. We created linear probability model specifications to estimate the probability of a female being visited by a CHW, including independent variables that might be more likely to make a female or a household more vulnerable. To account for village clustering, we also performed Wild cluster bootstraps (95% confidence interval) for the linear probability models to estimate SEs [30]. The outcome variables and covariates used in the analysis are described below.

#### Outcome variable

All eligible females were asked if they had received a home visit from a CHW in the past 6 mo. All females who reported receiving a CHW home visit were asked to report the name of their CHW. The 2 ASTUTE CHWs in each village were matched to the reported names using commonalities in the first name or last name and/or common nicknames or name misspellings of the CHW’s name. For example, if a female reported seeing a CHW named “Lizzie” and 1 of the CHWs in the village was named “Elizabeth,” then the female was matched to “Elizabeth” as her CHW. Females who saw both CHWs (40 females) were matched to the CHW that was closest to them geographically. There were 68 females who reported the names of CHWs that did not match any of the 2 CHWs in their village, likely due to CHWs operating within other community programs in the area. In addition, 90 females did not remember the name of their CHW. For this analysis, we excluded females who either did not remember the name of their CHW or who reported being visited by another person.

### Covariates

#### Household-level data

The household-level data included household size as a continuous variable, food security, wealth status, and distance. Food security was analyzed using the household food insecurity access scale, which was constructed using the standardized method described [31]. The categorical household food insecurity access scale indicator was used, which categorizes households as “Food Secure,” “Mildly Food Insecure Access,” “Moderately Food Insecure Access,” and Severely Food Insecure Access.” A wealth index was created using the demographic and health surveys wealth surveys’ index guidelines [32]. This method involved using principal component analysis to represent a composite measure of a household’s cumulative living conditions. Variables included in the wealth index estimation were ownership of household assets, makeup of the house (floor, roof, and wall), and type of electricity. Missing asset values were replaced with the value of “0.5,” as “0” was coded as not having the asset, and “1” was coded as having the asset. All “other” categories were translated into English (they were entered in Swahili) and either combined with existing categories or created into new categories. Wealth quintiles were generated from the wealth index, and the females and CHW wealth indices were computed together as 1 sample. Each household asset was normalized using its mean and standard deviation and created into a wealth quintile. The wealth quintile consisted of the “Poorest,” “Poorer,” “Middle,” “Richer,” and “Richest” categories.

#### Child-level data

Child age groups (0–3 mo, 3–9 mo, 9–12 mo, and 12–16 mo) were included in the models as binary categories. Females with a child with a history of malnourishment were determined per the child health card. Most children received a health card at the clinic, which tracked their height and weight and reported if either 1 fell below what was expected for children within the same age group.

#### Mother-level data

Female’s education level, religion, whether the female was in her first pregnancy, and polygamous marriages were all expressed as categorical covariates. Females’ age was kept as a continuous variable.

#### Distance

The distance was calculated as the distance between CHWs’ homes and females’ homes using the GPS coordinate data collected using ODK on the tablets. For females who did not report a CHW visit, the distance was calculated by matching those females to the nearest CHW in their village.

#### Homophily

In addition, we included variables to represent “homophily” to assess if CHWs tend to visit females who are more similar to them in age, education, wealth, and ethnic group. The age difference was determined by subtracting the female’s age from the age of the CHW. Education difference was calculated by subtracting the female’s level of education from the CHW’s level of education. For wealth difference, each wealth category was assigned a number (“1” was the poorest quintile, “2” was the poorer quintile, “3” was the middle quintile, “4” was the richer quintile, and “5” was the richest quintile) and the female’s wealth quintile number was subtracted from the CHWs wealth quintile number. Differences in ethnic group were a dichotomous variable, with females being assigned a “0” if they had a different ethnic group than their CHW and a “1” if they were the same ethnic group. Females who did not see a CHW were matched to the nearest CHW in their village to determine differences or similarities in age, education, wealth, and ethnic group.

### Qualitative analysis

Qualitative analysis was conducted using NVivo qualitative analysis software for coding and sorting of textual data. The qualitative analysis took place from June 2020 to August 2020, prior to the analysis of the quantitative data. Transcripts were analyzed thematically, using an inductive approach based on the principles of grounded theory and the constant comparative method [33]. Data were reviewed and analyzed descriptively. Open coding was used to identify codes, concepts, and themes in the data [34]. The codebook was developed during a process of line-by-line coding by 3 team members [34]. Each transcript was coded independently by 2 team members, and results were compared and integrated. Throughout the entire coding process, we continued to discuss and refine the codebook as an iterative process, and the codebook was approved by all members of the coding team.

### Ethical approval and consent

This study was approved by the National Medical Research Coordinating Committee of the Ministry of Health and Social Welfare (PROTOCOL NIMR/HQ/R.8a/Vol. IX/2905) in October 2018 and the Cornell University Ethics Review Board (Protocol #1611006801) in July 2018. For the quantitative data collection (census/survey and CHW survey), we obtained oral consent from participants. Written consent was obtained from the CHWs and CHW supervisors prior to completing the in-depth interviews.

## Results

### Quantitative results

**FIGURE 2:**
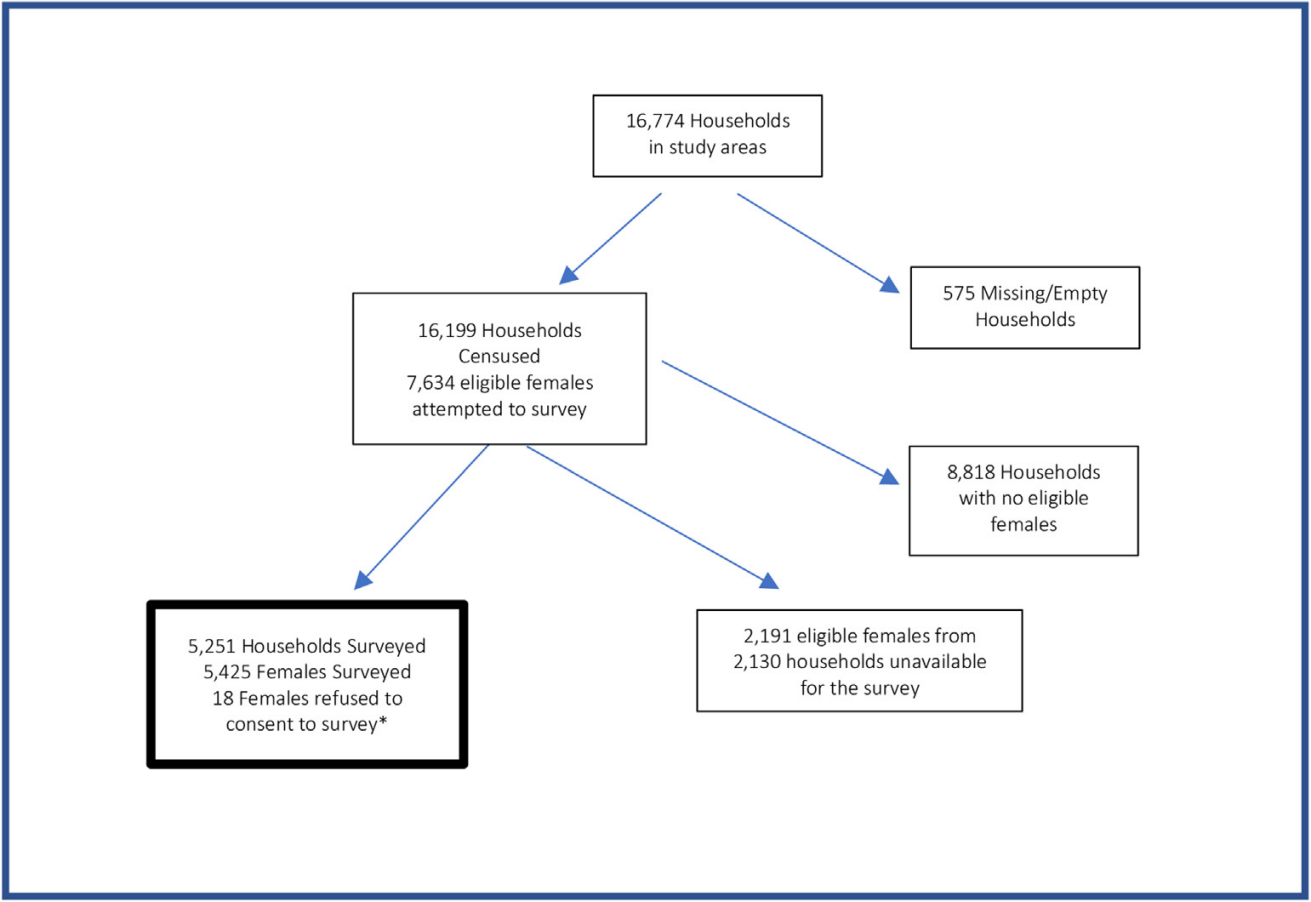
Study sample flow chart. ∗The 18 females who did not consent were part of the 5251 households where ≥1 other female agreed to complete the survey and provided household-level data.

For the census (stage 1), we visited 16,199 households. In the survey for eligible females (stage 2), we collected data from 5425 females from 5251 households. For the census/survey, we did not include 575 households (households where either no 1 was home at the time of the census or no one currently lived there) and 2191 females (females who were not present at the time we visited their household). In addition, 18 eligible females did not consent to the survey (Figure 2).

#### Participant characteristics

The demographic characteristics of females sampled are summarized in Table 3. Most females reported their households as food insecure and reported experiencing a household “shock” (e.g., financial hardship, death of a family member) in the past 6 mo. A majority of households included only 1 eligible female, but some households had 2, 3, or 4 eligible females. Although 58% of females had a primary school-level education, nearly 1 quarter had no schooling, and 29% were illiterate. Almost all females were married, Christian, and in a monogamous relationship. Most of the male partners in our sample had only primary school-level education.

**TABLE 3:** Household, child, female caregivers, and male partner demographics.

Variables	*n*	Mean (SD), *n* (%), or median (IQR)
Household characteristics		
Household size, mean (SD)	5249	5.7 (2.8)
Number of children under 18, mean (SD)	5250	3.3 (2.1)
Household food insecurity access scale, *n* (%)	5236	
Food secure		2188 (41.8)
Mildly food insecure		463 (8.8)
Moderately food insecure		1486 (28.4)
Severely food insecure		1099 (21.0)
Wealth quintile, *n* (%)	5251	
Poorest		1051 (20.0)
Poorer		1050 (20.0)
Middle		1050 (20.0)
Richer		1050 (20.0)
Richest		1050 (20.0)
Number of eligible females per household, *n* (%)	5425	
One female		5251 (96.8)
Two females		157 (2.9)
Three females		15 (0.3)
Four females		2 (0.0)
Child’s characteristics		
Child’s age in months, mean (SD)	4996	13.3 (8.7)
Child within ASTUTE priority age (3–9 mo), *n* (%)	4996	
No		3733 (74.7)
Yes		1263 (25.3)
Child’s malnourishment status, *n* (%)	4977	
No		4341 (87.2)
Yes		525 (10.6)
Missing card		111 (2.2)
Female caregivers characteristics		
Females’ age, mean (SD)	5425	27.3 (7.4)
Females’ education, *n* (%)	5386	
No schooling		1306 (24.3)
Preprimary		533 (9.9)
Primary		3101 (57.6)
Postprimary		48 (0.9)
Secondary O level		359 (6.7)
Postsecondary O level		26 (0.5)
Secondary A level		3 (0.1)
Postsecondary A level		1 (0.0)
University		9 (0.2)
Females’ literacy, *n* (%)	5399	
Cannot read at all		1549 (28.7)
Able to read only part of the sentence		529 (9.8)
Able to read the whole sentence		3295 (61.0)
No card with required language		24 (0.4)
Blind/visually impaired		2 (0.0)
Marital status, *n* (%)	5403	
Single		249 (4.6)
Married or has a partner		4793 (88.7)
Divorced/separated		293 (5.4)
Widowed		68 (1.3)
Polygamy, *n* (%)	4791	
No		4053 (84.6)
Yes		713 (14.9)
I don’t know		25 (0.5)
First pregnancy, *n* (%)	973	
No		769 (79.0)
Yes		204 (21.0)
Number of children under 2.5, mean (SD)	4662	1.1 (0.3)
Previous “shock” in past 6 mo, *n* (%)^1^	5397	
No		1705 (31.6)
Yes		3692 (68.4)
Receives TASAF (cash assistance), *n* (%)	5396	
No		5145 (95.2)
Yes		251 (4.7)
I don’t know		7 (0.1)
Number of years living in the village, median (IQR)	5396	7.0 (15)
Currently pregnant, *n* (%)	5402	
No		4425 (81.9)
Yes		975 (18.1)
I don’t know		2 (0.0)
Religion, *n* (%)	5403	
Traditional		7 (0.1)
Christianity		4130 (76.4)
Hinduism		2 (0.0)
Judaism		6 (0.1)
Muslim		572 (10.6)
Nonreligious		686 (12.7)
Knows the CHW in their village, *n* (%)	727	
No		158 (21.7)
Yes		569 (78.3)
Number of CHW visits in the past 6 mo	726	2.61 (3.0)
Length of time of last CHW visit (in minutes), median (IQR)	724	30 (30)
Partner characteristics		
Males’ age, mean (SD)	4451	33.3 (9.1)
Males’ education, *n* (%)	4700	
No schooling		795 (16.9)
Preprimary		398 (8.5)
Primary		3013 (64.1)
Postprimary		43 (0.9)
Secondary O level		335 (7.1)
Postsecondary O level		46 (1.0)
Secondary A level		24 (0.5)
PostSecondary A level		7 (0.2)
University		39 (0.8)

Abbreviations: ASTUTE, addressing stunting early in Tanzania; CHW, community health worker; IQR, interquartile range; SD, standard deviation; TASAF, Tanzanian Social Action Fund.

1 E.g., financial hardship, death, illness, or injury of a family member, poor harvests.

Characteristics of the sample of CHW are summarized in Table 4. Most CHWs were monogamous and married or in a partnership. There were considerable differences in education, food security, and wealth status between CHWs and females. CHWs were better off relative to the population that they served. Most CHWs were very experienced working as a CHW, and there was a wide range in the number of females that the CHWs reported to have seen through the ASTUTE program. We did not find any significant differences for number of females visited or distances traveled for home visits for male CHWs than female CHWs.

**TABLE 4:** Community health worker demographics and performance indicators

Variables (*n* = 66)^1^	Combined	Males	Females
		*N* = 32	*N* = 34
	Mean (SD) or *n* (%)	Mean (SD) or *n* (%)	Mean (SD) or *n* (%)
Age	45.2 (10.0)	47.5 (8.2)	43.1 (11.2)
Length of time living in the village	34.1 (16.2)	39.2 (15.9)	29.2 (15.3)
Marital status, *n* (%)			
Single	4 (6.1)	1 (3.1)	3 (8.8)
Married or has a partner	52 (78.8)	29 (90.6)	23 (67.7)
Divorced/separated	3 (4.6)	1 (3.1)	2 (5.9)
Widowed	7 (10.6)	1 (3.1)	6 (17.7)
Polygamy, *n* (%)			
No	39 (75.0)	23 (79.3)	16 (69.6)
Yes	13 (25.0)	6 (20.7)	7 (30.4)
Primary occupation, *n* (%)			
Sales and Services Work	66 (100)	32 (100)	34 (100)
Household food insecurity access scale, *n* (%)			
Food secure	32 (48.5)	12 (37.5)	20 (58.8)
Mildly food insecure	9 (13.6)	6 (18.8)	3 (8.8)
Moderately food insecure	17 (25.8)	9 (28.1)	8 (23.5)
Severely food insecure	8 (12.1)	5 (15.6)	3 (8.8)
Wealth quintile, *n* (%)			
Poorest	1 (1.5)	1 (3.1)	0 (0.0)
Poorer	5 (7.6)	2 (6.3)	3 (8.8)
Middle	5 (7.6)	4 (12.5)	1 (2.9)
Richer	16 (24.2)	7 (21.9)	9 (26.5)
Richest	39 (59.1)	18 (56.3)	21 (61.8)
Education, *n* (%)			
Primary	51 (77.3)	23 (71.9)	28 (82.4)
Postprimary	2 (3.0)	2 (6.3)	0 (0.0)
Secondary O level	11 (16.7)	6 (18.8)	5 (14.7)
Postsecondary O level	2 (3.0)	1 (3.1)	1 (2.9)
Religion, *n* (%)			
Christian	58 (87.9)	29 (90.6)	29 (85.3)
Muslim	6 (9.1)	2 (6.3)	4 (11.8)
Nonreligious	2 (3.0)	1 (3.1)	1 (2.9)
Literacy, *n* (%)			
Able to read only part of the sentence	2 (3.0)	1 (3.1)	1 (2.9)
Able to read the whole sentence	64 (97.0)	31 (96.9)	33 (97.1)
Household size	6.6 (3.0)	7.0 (3.1)	6.2 (2.8)
Number of children in households under 18	3.7 (2.3)	3.9 (2.3)	3.5 (2.2)
Number of children under 2	1.1 (0.5)	0.3 (0.5)	0.2 (0.4)
Length of time as a CHW (years)	14.2 (9.5)	15.0 (8.5)	13.4 (10.4)
Average distances traveled in a week for home visits (km), median (IQR)	8 (34)	8 (7)	12 (7)
Number of females seen per CHW in the past 6 mo (as reported by females), median (IQR)^2^	8 (12)	11 (17.5)	6.5 (9)
Length of time as a CHW (years)	14.2 (9.5)	15.0 (8.5)	13.4 (10.4)
Average reported distance traveled in a week for home visits (km), median (IQR)	8 (34)	8 (7)	12 (7)

Abbreviations: CHW, community health worker; IQR, interquartile range; SD, standard deviation.

1 Four villages only had 1 CHW.

2 Range is 0–47.

In the CHW/household matching, 727 (13.4%) of eligible households reported receiving a visit from a CHW, 4674 (86.2%) reported not receiving a visit from a CHW, and 24 (0.4%) reported that they were unsure if they had received a visit or not. Out of the 4674 females that were not seen by a CHW, 203 (3.7%) reported receiving a visit from a non-ASTUTE CHW. The percentage of both eligible and ASTUTE priority households visited was very low (Table 5).

**TABLE 5:** Percentage of priority and total eligible population that received a home visit from a **community health worker** in the past 6 **mo**

	Priority population^1^*n* (%)	Total eligible population^2^*n* (%)
Not seen by CHW	1700 (85.3)	4674 (86.2)
Seen by CHW	281 (14.1)	727 (13.4)
Unknown	11 (0.6)	24 (0.4)

Abbreviations: CHW, community health worker; TASAF, Tanzanian Social Action Fund.

1 Priority guidelines: households with a female caretaker of a child under the age of 30 mo that have a preschool child who was or has been mild or moderately undernourished, participate in TASAF (a government unconditional cash transfer program given to poor or vulnerable households, households with children or pregnant females, households with disabled family members), that have a mother in her first pregnancy, or households that have children aged 3–9 mo.

2 Total eligible: all households with a female caretaker of a child under the age of 30 mo.

**FIGURE 3:**
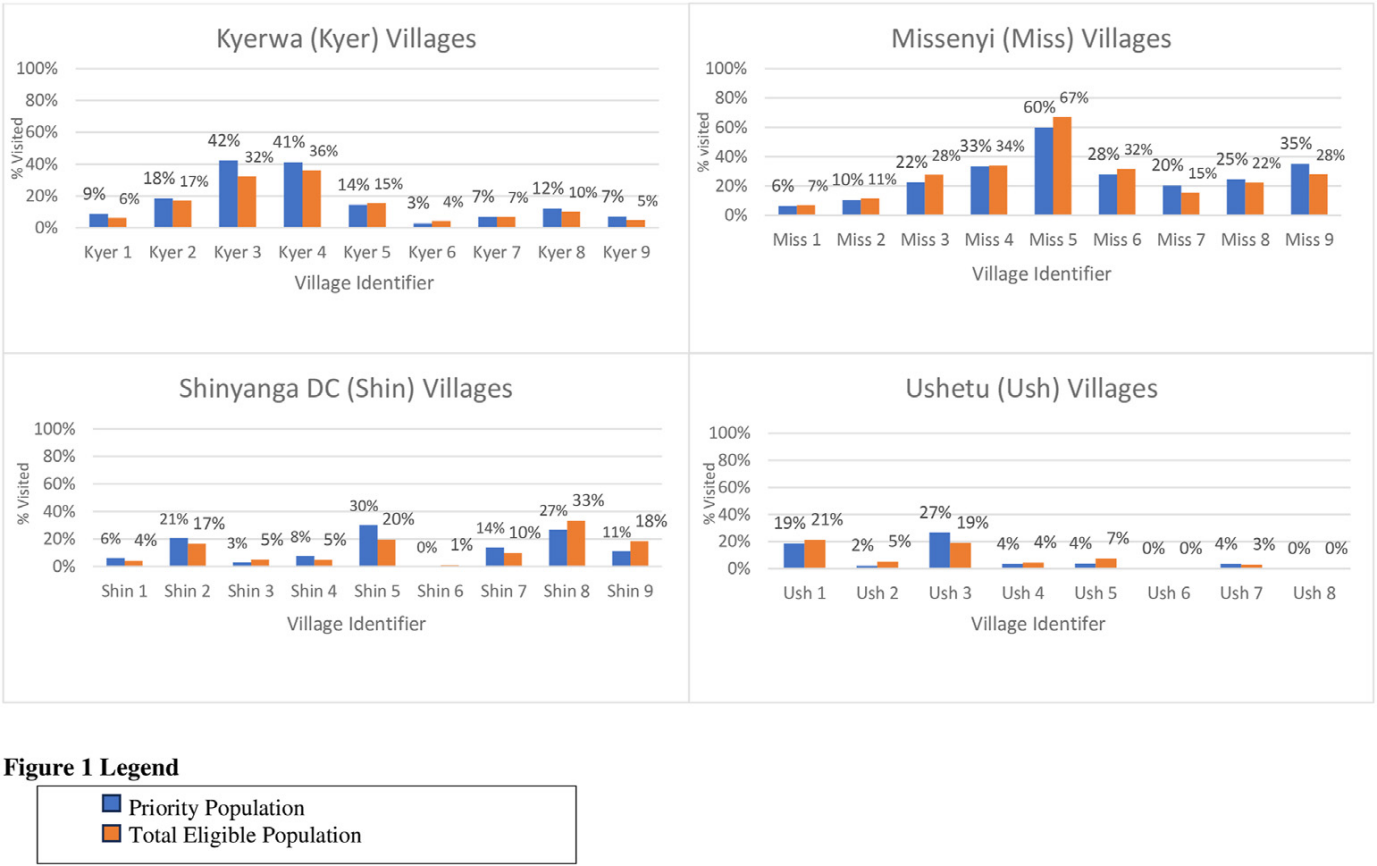
Percentage of the priority^1^ and total eligible population^2^ visited by village. TASAF, Tanzanian Social Action Fund. ^1^Priority guidelines: households with a female caretaker of a child under the age of 30 mo that have a preschool child who was or has been mild or moderately undernourished, participate in TASAF (a government unconditional cash transfer program given to poor or vulnerable households, households with children or pregnant females, households with disabled family members), that have a mother in her first pregnancy, or households that have children aged 3–9 mo. ^2^Total eligible: all households with a female caretaker of a child under the age of 30 mo.

**FIGURE 4:**
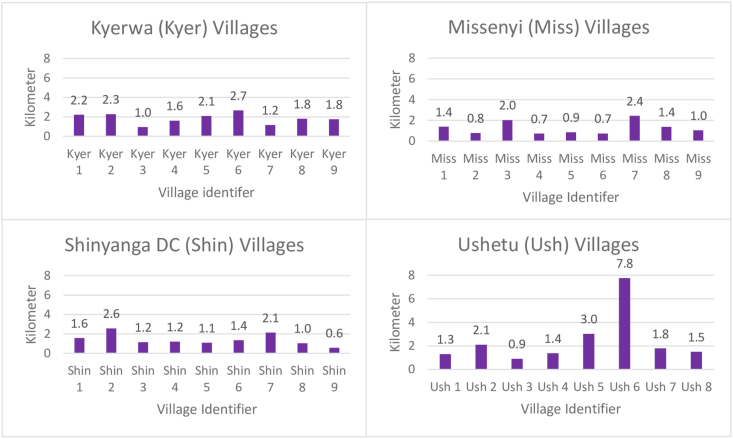
Average distance between CHW and females’ houses. CHW, community health worker.

**FIGURE 5:**
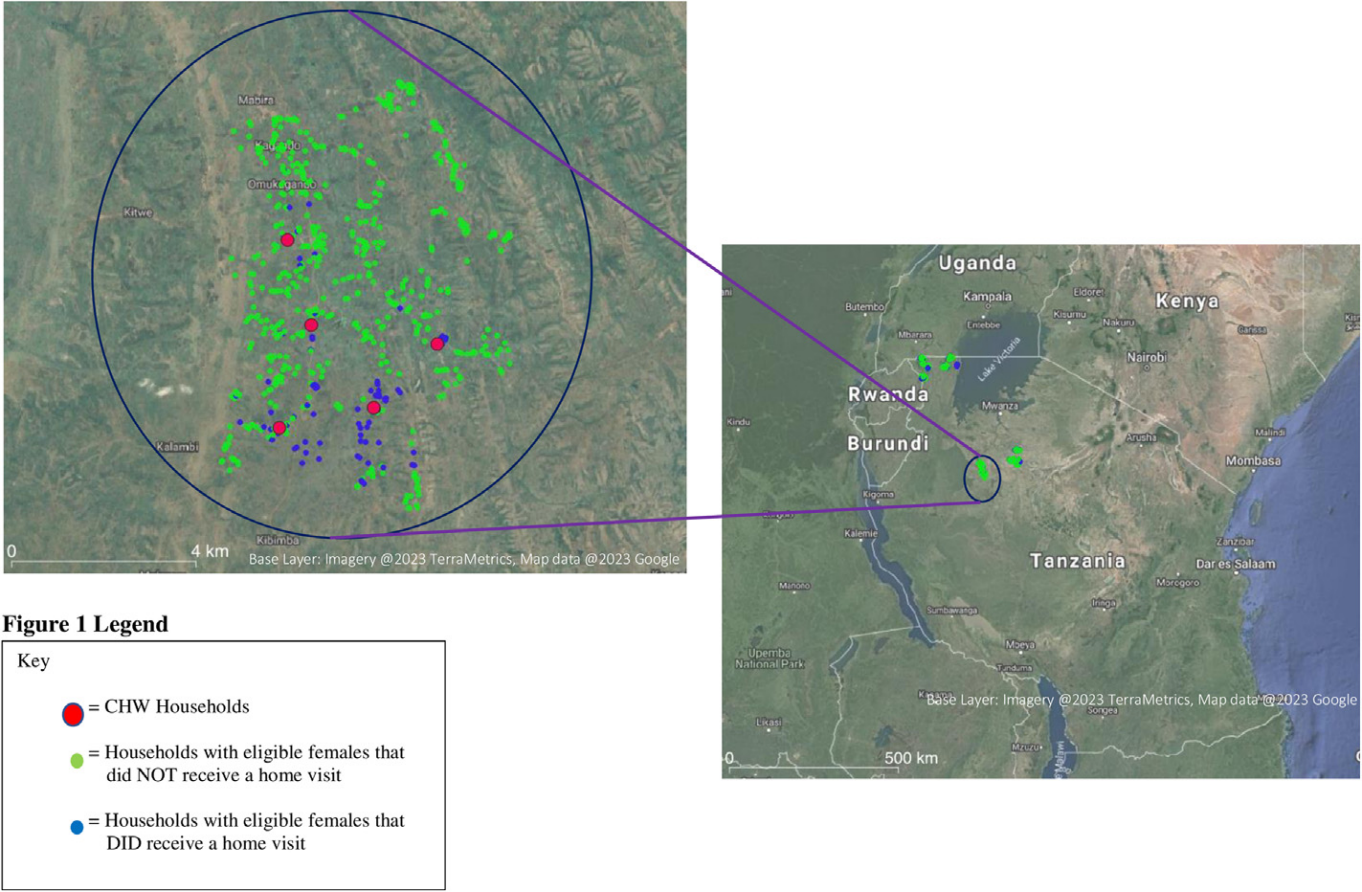
Geographic information system (GIS) mapping of a study ward site (3 villages). CHW, community health worker.

To assess village-level differences in coverage, we graphed the percentage of the priority and eligible population by village (Figure 3). We also graphed the average distances between CHW and eligible households per village (Figure 4). For both FIGURE 3, FIGURE 4, we separated the graphs by the 4 districts and used a village number instead of the name to maintain confidentiality. Figure 5 illustrates the mapping of CHWs and eligible households that were and were not visited in a single ward. There were disparities in the percentage of households visited per village, as well as patterns in the mappings with CHWs visiting households that were closer to them. Generally, the CHWs performed fewer household visits in villages with larger average distances between the CHW and eligible households.

First, we conducted bivariate analysis with several variables and the likelihood of receiving a home visit (Supplemental Table 1). After controlling for other variables in the linear probability models, many of the significant relationships in the bivariate analysis were attenuated (Table 6). Analysis was completed at the female level instead of the household-level, as there were multiple females in some households. We did not control for this household clustering as only 3.5% of females were the second, third, or fourth females in a household. Model 1 focused on the ASTUTE priority population variables, as these were the characteristics that CHWs were told to prioritize for home visits. In model 1, females with a child with a history of malnourishment were 4.4 percentage points (pp) less likely to receive a visit from a CHW. Females with a child aged 3–9 mo were 4.6 pp more likely to receive a visit from a CHW.

**TABLE 6:** Linear probability models: likelihood of a female being seen by a Community health worker

Variables			Model 1 b (SE)	Model 2 b (SE)	Model 3 b (SE)	Model 4 b (SE)	Model 5 b (SE)
ASTUTE priority	Female with child 3–9 mo		0.046 (0.016)∗∗	0.059 (0.020)∗∗	0.062 (0.020)∗∗	0.063 (0.019)∗∗	0.056 (0.020)∗∗
	Household participation in TASAF(cash assistance)		0.009 (0.023)	0.005 (0.023)	0.0003 (0.021)	–0.002 (0.019)	0.002 (0.019)
	Female in her first pregnancy		0.063 (0.128)	0.080 (0.132)	0.075 (0.130)	0.073 (0.132)	0.055 (0.126)
	Female with a child with a history of malnourishment		–0.043 (0.020)∗	–0.034 (0.019)	–0.034 (0.019)	–0.032 (0.019)	–0.032 (0.019)
Females participant characteristics	Female with child 0–3 mo			0.037 (0.018)∗	0.040 (0.018)∗	0.041 (0.018)∗	0.034 (0.019)
	Female with child 9–12 mo			0.021 (0.020)	0.019 (0.020)	0.018 (0.020)	0.012 (0.021)
	Female with child 12–16 mo			0.002 (0.014)	0.002 (0.014)	0.002 (0.013)	–0.001 (0.014)
	Female’s age			0.003 (0.001)∗∗	0.002 (0.001)∗∗	0.004 (0.002)	
	Female’s education			0.042 (0.014)∗∗	0.033 (0.013)∗∗	0.015 (0.028)	
	Females’ religion	Christianity (reference)					
		Other religion		0.061 (0.112)	0.058 (0.110)	0.058 (0.107)	0.058 (0.110)
		Islam		–0.021 (0.028)	–0.023 (0.026)	–0.023 (0.027)	–0.020 (0.027)
		Nonreligious		–0.053 (0.024)∗	–0.050 (0.022)∗	–0.047 (0.022)	–0.062 (0.023)∗
Household characteristics	Household size			–0.004 (0.002)	–0.003 (0.002)	–0.003 (0.002)	–0.002 (0.003)
	Food security (HFIAS)	Food Secure (reference)					
		Mildly Food Insecure		–0.020 (0.023)	–0.021 (0.023)	–0.024 (0.022)	–0.020 (0.021)
		Moderately food insecure		–0.026 (0.017)	–0.027 (0.016)	–0.028 (0.016)	–0.027 (0.014)
		Severely food insecure		–0.049 (0.021)∗	–0.049 (0.021)∗	–0.048 (0.020)∗	–0.049 (0.019)∗
	Wealth quintile	Richest (reference)					
		Richer		–0.013 (0.022)	–0.007 (0.023)	–0.013 (0.029)	
		Middle		–0.032 (0.026)	–0.019 (0.027)	–0.032 (0.042)	
		Poorer		–0.003 (0.023)	0.011 (0.024)	–0.007 (0.052)	
		Poorest		–0.050 (0.022)∗∗	–0.035 (0.021)	–0.060 (0.060)	
Distance (in km)					–0.031 (0.008)∗∗	–0.031 (0.009)∗∗	–0.032 (0.008)∗∗
Homophily variables	Age difference between CHW & female					0.001 (0.002)	–0.0001 (0.001)
	Education difference between CHW & female					–0.010 (0.015)	–0.015 (0.009)
	Wealth difference between CHW & female					0.007 (0.013)	–0.003 (0.005)
	Ethnic group difference between CHW & female					0.012 (0.018)	0.013 (0.018)

Abbreviations: ASTUTE, addressing stunting early in Tanzania; CHW, community health worker; HFIAS, household food insecurity access scale; SE, standard error; TASAF, Tanzanian Social Action Fund.

∗*P* < 0.05, ∗∗*P* < 0.01.

When controlling for other characteristics of females and households in model 2, mothers with children with a history of malnourishment were no longer statistically significantly less likely to receive a home visit. In addition to mothers with children aged 3–9 mo (a priority group), mothers with children aged 0–3 mo were also more likely to be seen by a CHW. Older and more educated females were also more likely to receive a home visit from a CHW. Females who were nonreligious were 5 pp less likely to be seen by a CHW than Christian females. In addition, households that were severely food insecure and in the poorest wealth quintile were each 5 pp less likely to be visited by a CHW.

In model 3, which included the distance as a covariate, the distance was highly significant, with females 3 pp/km less likely to be seen by a CHW the further away they lived from their CHW. Females with children 3–9 mo, females with children aged 0–3 mo, females with higher education, and older females were more likely to be seen by a CHW. Females who were nonreligious, in households that were severely food insecure, and/or in the poorest wealth category were still significantly less likely to receive a home visit by a CHW.

In model 4, which included the set of homophily variables, the age difference between the CHW and the mother, education difference, wealth difference, and difference in ethnicity did not affect the CHW targeting of households. After removing the household and mother-level variables that are collinear with the homophily variables in model 5 (i.e., female’s age, female’s education level, and household wealth status), these homophily variables still had no effect on whether a female received a home visit.

For all model specifications, the Wild cluster bootstraps (Supplemental Table 2) provided identical results to the original analysis. We also graphed the percentage of eligible females who did not receive a visit by the village to see if there were village-level differences (Supplemental Figure 1). Because our ability to interview eligible females seemed to differ by village, we re-analyzed model 4 with dummy variables for each village (Supplemental Table 3). We saw little difference between the 2 models, indicating that sample selection is unlikely to bias our results.

Overall, our quantitative analyses indicate that females with a child aged 3–9 mo were more likely to receive a home visit from a CHW, and this was not sensitive to model specification. Females with a child aged 0–3 mo, females in severely food insecure households, and females living further away from the CHW were less likely to receive a home visit, regardless of model specification. Females with less education, younger females, and nonreligious females (as compared to Christian females) were less likely to receive home visits by a CHW in model specifications 2 and 3. Females from households that were severely food insecure were less likely to receive a home visit by a CHW in specification 2, but this relationship is attenuated with the addition of distance as a covariate. There was no association of households receiving TASAF (cash assistance) or females in their first pregnancy with the likelihood of receiving a home visit, and this wasn’t sensitive to the model specification. If the female had a child with a history of malnourishment or was from a poorer household, they were less likely to receive a visit, but the significance was dependent on the model (model 1 only). The statistical significance of females with a child with a history of malnourishment being less likely to receive a visit from a CHW was just slightly >0.05 in models 2–4.

### Qualitative results

Several common themes were highlighted by the CHWs and supervisors in interviews. We have organized the qualitative results section to discuss *1*) convergent themes supported by the quantitative data and *2*) themes that either diverge from or are unsupported by the results of the quantitative data. Representative quotes are below in Table 7.

**TABLE 7:** Community health worker interview quotes illustrating key themes related to targeting home visits

	Themes	Quotes
Convergent themes^1^	Challenges in visiting females with low levels of education	“It is difficult because there are people who haven’t attended school, then you need to spend a long time educating that family; that means you just can’t get there today to educate them and leave; you need to educate them often.” -CHW-86838d1b, 38M
	Challenges visiting households who are poor or food insecure	“The challenge which I face is that when you tell her of what she should eat, she will tell you that it would have been better if you brought those foods which you are telling her to eat. I tell her that we are only offering education as we are also being taught, and she will say no, you are being paid something, but you don’t want to give it to us, we keep on insisting that we don’t get anything otherwise, would be giving it to them.”- CHW-3534c95d, 55F
	Challenges due to village size/geography	“There are challenges on how to reach the targeted ones, especially those who are far, for example as for you have seen how far it is from 1 household to another…there are 2 CHWs in every village, and you may find a single village has 7 hamlets (administrative subdivision indicating village size), so they will divide 3 and a half hamlets each. If you consider the distance, sometimes it becomes an obstacle to reach the participants with the current target. During heavy rains, if you will look, they are coming from 1 place to another while carrying their materials; you may find sometimes the rain becomes an obstacle, or it might rain while he or she is on their way, and the materials might be destroyed.” -CHW Supervisor-4
		“I think the project can support by giving me another CHW to assist me because I am all alone and I have 13 hamlets (administrative subdivision indicating village size); other people can’t have access to the program.” -CHW-d9abcd64, 36F
	Targeting first-time pregnant females	“You know it is hard to identify people with first pregnancies…you just can’t go from nowhere to tell her that I was told that you are pregnant; she will ask you who told you that I am pregnant. So you will just have to go slowly and tell her that I have come to see you, and mostly, I usually ask have you started to attend clinic?, she would ask which clinic is that, then I would tell her that you seem (to be pregnant) so I am advising you to attend clinic early. She would tell you, ‘So many people already know?’ …. It is difficult to identify the first pregnancy; you may see it when it is 7 or 6 mo.” - CHW-725b9b67, 43M
Divergent themes^2^	Random targeting	“We are going from 1 household to another…I go with my fellow CHW; we are visiting 1 household after another without caring this is a pregnant or postpartum female or a female with a child under 2 y child, so we are visiting all households to offer general education.” – CHW-78adc9e6, 48F
	Visiting migrants	“Yes, for example, the people in rural areas they have a problem of poor understanding, but they do respond well, for example in (retracted) village, you find migrants, you would find a person knows neither Swahili (Tanzania’s national language) or Haya (local area specific language), so for that person to understand you, you will need a lot of time and a bunch of signs.”-CHW-63a4943d, 44M
	Importance of targeting males	“The first challenge, as I have said earlier, males have the biggest responsibility in making sure the nutrition is available in the family; they can’t be found to participate in the home visit discussions, only a very small percentage, so that is the first challenge, and it is the main one.” - CHW Supervisor-2

Abbreviations: CHW, community health worker; M/F, male/female.

1 Convergent themes are themes supported by quantitative data.

2 Divergent themes are themes that either oppose or are unsupported by the quantitative data.

#### Convergent themes

CHWs and supervisors mentioned many general barriers to targeting females for home visits. These perspectives help to explain some of the trends seen in the quantitative data analysis. For example, physical barriers such as large village size and poor weather were mentioned as major challenges. Despite wide variation in the size of villages, 2 CHWs were trained for each village. Some villages only had 1 CHW because the other had quit or left, leaving 1 person responsible for the entire village. The CHWs lacked access to weather gear like umbrellas and rain boots, which made travel very difficult or impossible during the rainy season. The CHWs mentioned that not having access to transportation made household visits difficult for large villages or villages that are heavily affected by rainfall. Although the CHWs were paid a stipend for completing the home visits, many of them considered the stipend too low. Many CHWs discussed wanting an increase in their stipend or the addition of a separate transportation allowance. These results align with the quantitative results, as households that were further away from their CHW were less likely to receive a home visit.

CHWs also shared the social difficulties of visiting females with low education and females in households that are poor or food insecure. Many of the CHWs felt that it took too long to explain the health and nutrition education they were promoting if the mother was not educated or had poorer levels of understanding. They also felt it was difficult to visit very poor households because they were only offering education during the home visits. CHWs discussed the challenges of providing education without offering any food or money to help households implement recommended health and nutrition behaviors. This was especially challenging for CHWs visiting households with children who were very malnourished. In the quantitative results, we saw that TASAF households (which were very poor) were less likely to receive a visit from a CHW, even though prioritized in the guidelines. Females with higher levels of education were more likely to receive a visit from a CHW, and females in households that were severely food insecure were less likely to receive a home visit.

CHWs also mentioned difficulties in targeting first-time pregnant females or pregnant females in general. Although CHWs wanted to target pregnant females for maternal health and nutrition education, they often found it difficult to know when females were pregnant. CHWs were not able to determine who was pregnant until the female started showing or started attending clinic visits, which could be late in the pregnancy. It was especially hard for CHWs to keep track of females in isolated or remote areas of the village. Although this was a program priority population, we saw in the quantitative results that females in their first pregnancies did not seem more likely to receive a home visit by a CHW.

#### Divergent themes

In addition to the themes discussed above, the CHWs reported other themes related to the targeting of households. Some CHWs discussed random targeting of households or would just visit any households with a child or pregnant female. The CHWs also discussed the challenges of visiting migrants from other countries due to language and cultural barriers. Migrants were especially common in border towns. We did not see this directly in the homophily results (migrants generally were a different ethnic group than the village CHW, and we did not see that CHWs were more or less likely to visit those different than them). However, these migrants were also most likely to live very far from the CHW, which may be explained by the distance results.

Lastly, although this wasn’t measured in the quantitative data, many CHWs also talked about the importance of targeting males during home visits, as they were the ones responsible for providing food for the family. CHWs often mentioned they would try to visit females when they knew the husbands were home. It is possible that this may also play a role in who is more or less likely to receive a home visit from a CHW.

## Discussion

Historically, health care systems in the Global South have relied on CHWs due to shortages in healthcare workers and, in some cases, as a horizontal learning approach. CHWs have the potential to positively impact their communities and play a role in motivating positive health and nutrition behaviors. CHWs are uniquely positioned to link their communities to the health care system [12]. However, heavy workloads are a major challenge for CHWs in the Global South [35,36]. They may be seen as full-time paid government workers with a regular salary by community members, which is often not the case [37]. CHWs are not always able to meet the expectations placed on them, as they may be tasked with managing many different responsibilities [38]. Given these constraints, CHWs working in large geographic areas must make important decisions about whom they prioritize for visits. Assessing barriers and contributors to the success of CHWs is helpful for identifying considerations for programs utilizing CHWs.

In our study, CHWs’ overall coverage of eligible households was very low. Very few females reported receiving a visit from a CHW and were able to be matched to a CHW in their village (727 or 13.4%). The number of females reporting visits by a CHW over the past 6 mo indicates that CHWs completed ∼0.5 visits a week on average. This would be ∼6 visits a quarter, much less than the average number of visits per quarter reported by CHWs (24 visits) and the program expectations for CHWs (72 visits/quarter). Given this low coverage of households, we roughly estimated the time needed if CHWs could visit all eligible households or all ASTUTE priority households, i.e., households with a female caretaker of a child under the age of 30 mo that: have a preschool child who was or has been mild or moderately undernourished, a first-time pregnant female, a child aged 3–9 mo, or that participate in TASAF. The CHWs all worked full-time in other jobs (sales and services work). The average distance between CHW and female households was 1.78 ± 1.46 km (range: 0.002–11.543), requiring a total roundtrip of 3.6 km. Given holidays or other work constraints, if CHWs were to complete ≥2 visits a week (instead of the 6 required by the ASTUTE program), it would take the CHWs over a year to visit all the eligible households in their village, especially because new females would become pregnant in this time and add to the number of households. If the CHWs only visited the priority households and conducted 2 visits per week, it would take them ∼6 mo to visit all 1992 priority households once.

The effect of distance to households on the likelihood of receiving a home visit by a CHW was highly significant in all model specifications in the quantitative data and 1 of the biggest constraints reported by CHWs. The CHWs discussed the challenge of traveling far distances in bad weather and many reported that it simply was not possible to cover their village and provide home visits to everyone who needed one. Even when CHWs are highly motivated and are offering their services to improve their community, weather, time, and village size remain challenges for them in reaching community members. Other research has also found that high workloads and large distances between houses or work sites are barriers for CHWs in programming [39].

The CHWs in this program referred to themselves as volunteers, and they had other responsibilities that prevented them from being able to travel far distances for home visits. Even though they were paid a small stipend, they reported that they often went long periods of time without payment due to program delays. This likely influenced the number of home visits completed, as CHWs needed to perform their regular work activities. They were not paid a travel stipend for visiting households that were far away. Historically, payment to CHWs was considered unsustainable due to lack of funding [40,41]; however, there is now consensus that CHWs should be paid for economic and gender equity, as a majority of CHWs worldwide are females [40,42]. Payment should also be consistent with the expected workload and responsibilities, as lack of payment can contribute to a lack of motivation to complete program tasks, economic well-being, and psychosocial distress [39,40,[43], [44], [45]]. Payment, transportation support, and consistent management and supervision are important for scaling up and sustainability of CHW programming [39,42,46].

Other variables such as food security, age, and education were also significant in model specifications that included distance. This suggests some CHW bias in choosing whom to visit, even after accounting for distance. In the qualitative interviews, the CHWs commonly discussed how the program provided only health and nutrition education but did not address participants’ resource constraints and the challenges of visiting females who were less educated because it was harder for them to understand the health messages the CHWs were providing. They stated they had difficulty visiting poorer/food insecure households because the mothers or caretakers expected money or food from them. Although interventions targeting the poor often have a greater capacity to improve child health by identifying those most at risk [47], CHWs found it challenging to visit households they knew were not able financially to implement recommended behaviors such as consuming animal source foods. Although CHWs are often utilized because of their familiarity and credibility within their own communities, our results imply that there may be class structures that prevent CHWs from connecting with those less educated and less resourced than them. In interviews, CHWs noted visits to less educated females took longer, which may not be feasible when the CHWs already have a high workload and limited time.

CHWs were less likely to visit females with children with a history of malnourishment, in direct contradiction to the program guidelines. The CHWs may have had difficulty identifying which households met the priority criteria, which could have led to low coverage. Other studies of CHWs have found that they can be effective in identifying pregnant females before they attend health care visits [15]. However, in our study, CHWs either did not identify females in their first pregnancy (or any pregnancy) or did not prioritize visiting them. In the qualitative data, CHWs discussed the difficulty in determining which females were pregnant.

The homophily variables we were able to measure did not seem to impact targeting, as CHWs were not more likely to visit females who were more like them in terms of age, wealth, ethnic group, or education. Although the literature has shown people are more likely to visit or interact with people like them [23,24], we did not see this in our results. Although age, wealth status, and education affected the likelihood of receiving a visit from a CHW, the degree of similarity between CHWs and females did not predict the likelihood of receiving a home visit. For example, although CHWs were 3 pp/y more likely to see older females, the age difference between the CHW and the mother did not matter, such that a mother 5 y younger than a CHW is equally likely to be visited as a female who is 5 y older. It is possible that there may be other factors influencing homophily, for example, some unmeasurable characteristics or sociocultural factors that influence whom a CHW may choose to visit.

This study highlights the difficulty of relying on volunteers (or workers receiving minimal payment), even if they are working in their community, as they are likely to have other responsibilities and obligations. It may be more effective (although costly) to employ a sufficient number of CHWs as full-time staff members paid a livable wage so they can devote enough time to implement projects successfully. Other programs have found success in compensating CHWs based on their performance [48,49]. CHWs also need training on delivering nutrition counseling in “challenging” situations, specifically community members who may have a poorer understanding or be food insecure. In addition, targeting can be difficult, so it may be necessary to support CHWs and supervisors with mechanisms for identifying priority households for home visits, such as a system for tracking the health and nutrition of females and children in a community, for these programs to be most effective. Understanding the local context and what is needed to support program staff is important for influencing program design to ensure feasibility.

Other ways to strengthen the approach involve streamlining health programming. Other studies have found that CHWs can be better supported when given well-defined, specific roles [37]. Limiting the target population and catchment area might also make the program targeting more successful. Allowing CHWs to focus on a smaller number of people might be more effective than trying to target a larger number of people.

In the current era of technology and COVID-19, digital services have also been explored to ease the work burden of CHWs. Although digital services (mobile applications, phones, and tablets) can help to expand access and reach of programming, many challenges, such as poor digital literacy of CHWs, as well as lack of available funding, are present [50]. Although mobile technology can reduce workload and improve CHW supervision and reporting, often, there are internet connectivity and technological challenges that may affect programming for CHWs and their clients [51]. Mobile health interventions or applications have the potential to reduce the need for travel, which can expand CHW reach and improve their quality of care [52,53]. However, CHWs need to be supported with training and proper communication to implement these types of mobile health interventions [54]. Mobile health interventions also need to be culturally tailored to the setting in which they are operating [54].

Ultimately, continuing to assess CHW workload, involving CHWs in program planning, and giving CHWs opportunities to discuss their challenges may help balance the expectations of CHWs and programs [35,55]. Given the differing contexts across the Global South, CHW’s involvement in program planning is important for managing expectations. Ongoing training for CHWs is also necessary to frequently discuss strategies for visiting more difficult households, but training is often variable [56].

Our study had significant strengths. This was a large, mixed-methods research study that covered many villages. We were able to capture diversity in females, CHWs, and village characteristics, which allowed for more validity in our results. We interviewed CHWs, which helped to explain the quantitative results. We assessed other barriers for CHWs besides the size of the population and considered homophily and a range of demographic characteristics. To date, we are unaware of studies of this size that have assessed how CHWs target females in community programming. Lastly, we also collected GPS data from CHWs and community females and controlled for distance in our models alongside more commonly measured variables.

Our study also had some limitations. Our research team also was not able to survey ∼30% of the population across all villages. Some of these females were not available due to community events such as weddings and funerals. Even with our 10 full-time data collectors, we were unable to locate all eligible and priority females; however, the missing data was random and unlikely to bias our results. Our team was able to visit very remote or far households that would not be feasible for CHWs, especially without any transportation. Even if we were able to capture these missing females, this is likely to make the percentage of females visited even lower, as there are some females who may always be difficult to reach by CHWs. In addition, because the ASTUTE program was focused on the first 1000 d, we also focused this study on program-eligible participants. We were not able to capture how many ineligible females or households (for example, a household with a 5-y-old child) received a home visit from a CHW. A limitation on generalizability is related to region and district site selection. Our study sites had higher reported CHW home visits than the other ASTUTE rural regions and districts, as we wanted to conduct this research in areas where the program was operating reasonably well. These results are “best case scenario,” capturing “high” performing areas.

The CHWs were central to ASTUTE programming. Although they had local supervisors, the ASTUTE CHWs worked relatively independently to select households for home visits in their communities. Ultimately, CHWs have the potential to play a role in improving the practice of health behaviors in their community. However, their success will depend on their ability to target and visit the households that need it the most. We found that CHWs were unable to reach some of the most vulnerable populations due to barriers such as long travel distances. CHWs were also uncomfortable interacting with certain populations, such as poorer households or households where the mother was less educated. Future interventions that intend to draw on CHW-type workers in settings similar to our study, might well benefit from considering how best to staff and support workers when potential clients are dispersed over large geographic areas. They should also consider how best to work with populations that are poor and where caregivers lack formal schooling. Involving CHWs in all aspects of programming and providing needed resources and support can potentially increase the effectiveness of these types of interventions.

## Author contributions

The authors’ responsibilities were as follows – IO, JH, RBK, KD: designed the research; IO, RK: conducted the research; IO, JH, KD: analyzed the data; IO, JH, RBK, KD: wrote the article. IO had primary responsibility for the final content; and all authors: read and approved the final manuscript.

## Conflict of interest

The authors report no conflicts of interest.

## Funding

This work was funded by United Kingdom Aid from the United Kingdom government through the Addressing Stunting in Tanzania Early project. Our funders had no involvement in the study design, in the collection, analysis, and interpretation of data, in the writing of the report, and in the decision to submit the article for publication.

## Data availability

The data underlying the results presented in the study are available from the corresponding author after receiving written approval from the Tanzania Food and Nutrition Center (info@tfnc.go.tz or https://www.tfnc.go.tz/contactus).

## Figures and Tables

**Figure:**