# Supplementary material for: Community Health Workers’ Targeting of Women For Health and Nutrition Home Visits in Rural Tanzania: A Mixed-Methods Study
Source: Curr Dev Nutr. 2024 May 18;8(6):103780. doi: 10.1016/j.cdnut.2024.103780 (PMC11208947; doi:10.1016/j.cdnut.2024.103780)
Supplement: Multimedia component 1 [file mmc1.docx]

Supplementary Table 1: Bivariate Analysis of household characteristics by whether they received a home visit. (Households categorized as receiving a visit if females said they were visited and were able to name the CHW)

| Variables | n | Did not receive a home visit | Received a home visit | Mann Whitney/Chi-Square/Fisher exact | |
| --- | --- | --- | --- | --- | --- |
|  |  | mean (sd)  or n (%) | mean (sd)  or n (%) | p-value | |
| **Household Characteristics** | | | | |  |
| Household size, mean (sd) | 5,249 | 5.7 (2.8) | 5.6 (2.4) | 0.6764 | |
| Number of children under 18, mean (sd) | 5,250 | 3.3 (2.1) | 3.3 (1.9) | 0.3713 | |
| Household food insecurity access scale, n (%)  Food Secure  Mildly Food Insecure  Moderately Food Insecure  Severely Food Insecure | 5,236 | 1,834 (40.6)  393 (8.7)  1,300 (28.8)  992 (22.0) | 351 (49.4)  69 (9.7)  184 (25.9)  107 (15.1) | <0.0001 | |
| Wealth Quintile, n (%)  Poorest  Poorer  Middle  Richer  Richest | 5,251 | 965 (21.3)  893 (19.7)  925 (20.4)  898 (19.8)  850 (18.8) | 91 (12.8)  154 (22.6)  131 (18.4)  152 (21.3)  186 (26.1) | <0.0001 | |
| **Child’s Characteristics** | | | | |  |
| Child’s age in months, mean (sd) | 4,996 | 13.5 (8.7) | 12.00 (8.6) | <0.0001 | |
| Child within ASTUTE priority age (3-9mo), n (%)*  No  Yes | 4,996 | 3,257 (75.9)  1,037 (24.2) | 470 (67.6)  225 (32.4) | <0.0001 | |
| Child’s malnourishment status, n (%)*  No  Yes | 4,977 | 3,707 (88.7)  474 (11.3) | 628 (92.6)  50 (7.4) | 0.002 | |
| **Female Caregiver Characteristics** | | | | |  |
| Females’ age, mean (sd) | 5,425 | 27.2 (7.4) | 27.9 (6.7) | 0.0001 | |
| Females’ education, n (%)  No Schooling  Pre-primary  Primary  Post-Primary  Secondary O Level  Post-Secondary O Level  Secondary A level  Post-Secondary A Level  University | 5,386 | 1,179 (25.3)  482 (10.4)  2, 630 (56.5)  41 (0.9)  292 (6.3)  23 (0.5)  3 (0.1)  1 (0.0)  8 (0.2) | 124 (17.2)  51 (7.1)  469 (65.0)  7 (1.0)  67 (9.3)  3 (0.4)  0 (0.0)  0 (0.0)  1 (0.1) | <0.0001 | |
| Females’ Literacy, n (%)  Cannot read at all  Able to read only part of the sentence  Able to read the whole sentence  No card with required language  Blind/Visually impaired | 5,399 | 1, 406 (30.1)  465 (10.0)  2, 772 (59.4)  23 (0.5)  2 (0.0) | 140 (19.3)  64 (8.8)  521 (71.8)  1 (0.1)  0 (0.0) | <0.0001 | |
| Marital Status, n (%)  Single  Married or has partner  Divorced/Separated  Widowed | 5,403 | 212 (4.5)  4,141 (88.7)  260 (5.6)  58 (1.2) | 37 (5.1)  648 (89.1)  32 (4.4)  10 (1.4) | 0.553 | |
| Polygamy, n (%)  No  Yes | 4,791 | 3,477 (84.4)  643 (15.6) | 573 (89.3)  69 (10.8) | 0.001 | |
| First Pregnancy, n (%)*  No  Yes | 973 | 692 (78.6)  188 (21.4) | 77 (82.8)  16 (17.2) | 0.339 | |
| Number of children under 2.5, mean (sd) | 4,662 | 1.09 (0.3) | 1.10 (0.3) | 0.4551 | |
| Previous “shock” in past 6 mo?, n (%)  No  Yes | 5,397 | 1,428 (30.6)  3,237 (69.4) | 276 (38.0)  451 (62.0) | <0.0001 | |
| Receives TASAF (Cash assistance), n (%)*  No  Yes | 5,396 | 4,447 (95.3)  218 (4.7) | 694 (95.5)  33 (4.5) | 0.873 | |
| Number of years living in village, median (IQR) | 5,396 | 7 (16) | 8 (15) | <0.0001 | |
| Currently pregnant, n (%)  No  Yes | 5,402 | 3,786 (81.1)  882 (18.9) | 634 (87.2)  93 (12.8) | <0.0001 | |
| Religion, n (%)  Traditional  Christianity  Hinduism  Judaism  Muslim  Non-religious | 5,403 | 5 (0.1)  3,523 (75.4)  2 (0.0)  5 (0.1)  496 (10.6)  640 (13.7) | 2 (0.3)  603 (82.9)  0 (0.0)  1 (0.1)  76 (10.5)  45 (6.2) | <0.0001 | |
| **Partner Characteristics** | | | | |  |
| Men’s age, mean (sd) | 4,451 | 33.20 (9.1) | 33.81 (8.6) | 0.0183 | |
| Men’s education, n (%)  No Schooling  Pre-primary  Primary  Post-Primary  Secondary O Level  Post-Secondary O Level  Secondary A level  Post-Secondary A Level  University | 4,700 | 733 (18.1)  357 (8.8)  2,552 (62.9)  36 (0.9)  278 (6.9)  40 (1.0)  21 (0.5)  5 (0.1)  36 (0.9) | 61 (9.6)  41 (6.4)  459 (71.8)  7 (1.1)  57 (8.9)  6 (0.9)  3 (0.5)  2 (0.3)  3 (0.5) | <0.0001 | |

*ASTUTE priority Guidelines

**Supplementary Table 2: p-Values for Wild Cluster Bootstrap for Linear probability models (Table 9)**

| **Variables** | | | **Model 1** | **Model 2** | **Model 3** | **Model 4** | **Model 5** |
| --- | --- | --- | --- | --- | --- | --- | --- |
| **ASTUTE Priority** | Female with child 3-9mo | | 0.005 | 0.001 | 0.0001 | 0.0001 | 0.002 |
|  | Household participation in TASAF (Cash assistance) | | 0.702 | 0.835 | 0.986 | 0.919 | 0.918 |
|  | Female in her first pregnancy | | 0.639 | 0.562 | 0.581 | 0.599 | 0.683 |
|  | Female with child with history of malnourishment | | 0.041 | 0.093 | 0.099 | 0.108 | 0.109 |
| **Females’ Participant Characteristics** | Female with child 0-3mo | |  | 0.051 | 0.031 | 0.028 | 0.085 |
|  | Female with child 9-12mo | |  | 0.300 | 0.354 | 0.351 | 0.563 |
|  | Female with child 12-16mo | |  | 0.894 | 0.881 | 0.863 | 0.955 |
|  | Females’ Age | |  | 0.002 | 0.005 | 0.079 |  |
|  | Females’ Education | |  | 0.006 | 0.025 | 0.607 |  |
|  | Females’ Religion | Christianity(ref) |  |  |  |  |  |
|  |  | Other religion |  | 0.616 | 0.615 | 0.623 | 0.631 |
|  |  | Islam |  | 0.471 | 0.419 | 0.452 | 0.470 |
|  |  | Non-religious |  | 0.035 | 0.040 | 0.061 | 0.019 |
| **Household Characteristics** | Household size | |  | 0.135 | 0.247 | 0.267 | 0.582 |
|  | Food Security (HFIAS) | Food Secure(ref) |  |  |  |  |  |
|  |  | Mildly Food Insecure |  | 0.410 | 0.380 | 0.287 | 0.365 |
|  |  | Moderately Food Insecure |  | 0.130 | 0.107 | 0.102 | 0.075 |
|  |  | Severely Food Insecure |  | 0.033 | 0.036 | 0.030 | 0.016 |
|  | Wealth Quintile | Richest (Ref) |  |  |  |  |  |
|  |  | Richer |  | 0.576 | 0.757 | 0.683 |  |
|  |  | Middle |  | 0.244 | 0.495 | 0.495 |  |
|  |  | Poorer |  | 0.988 | 0.657 | 0.896 |  |
|  |  | Poorest |  | 0.015 | 0.107 | 0.367 |  |
|  | Distance (in km) | |  |  | 0.0005 | 0.0007 | 0.0002 |
| **Homophily Variables** | Age difference between CHW & female | |  |  |  | 0.504 | 0.992 |
|  | Education difference between CHW & female | |  |  |  | 0.587 | 0.134 |
|  | Wealth difference between CHW & female | |  |  |  | 0.648 | 0.585 |
|  | Ethnic group difference between CHW & female | |  |  |  | 0.536 | 0.490 |

**Supplementary Table 3: Linear Probability Model 5 with village dummy variables**

| **Variables** | | | **Dummy Variable Model**  **b (SE)** |
| --- | --- | --- | --- |
| **ASTUTE Priority** | Female with child 3-9mo | | 0.057 (0.018)** |
|  | Household participation in TASAF (Cash assistance) | | -0.008 (0.022) |
|  | Female in her first pregnancy | | 0.062 (0.113) |
|  | Female with child with history of malnourishment | | -0.024 (0.016) |
| **Females’ Participant Characteristics** | Female with child 0-3mo | | 0.034 (0.016)** |
|  | Female with child 9-12mo | | 0.018 (0.020) |
|  | Female with child 12-16mo | | 0.012 (0.013) |
|  | Females’ Age | |  |
|  | Females’ Education | |  |
|  | Females’ Religion | Christianity(ref) |  |
|  |  | Other religion | 0.083 (0.098) |
|  |  | Islam | -0.002 (0.021) |
|  |  | Non-religious | -0.019 (0.012) |
| **Household Characteristics** | Household size | | 0.0001 (0.002) |
|  | Food Security (HFIAS) | Food Secure(ref) |  |
|  |  | Mildly Food Insecure | -0.027 (0.018) |
|  |  | Moderately Food Insecure | -0.023 (0.010)* |
|  |  | Severely Food Insecure | -0.049 (0.016)** |
|  | Wealth Quintile | Richest (Ref) |  |
|  |  | Richer |  |
|  |  | Middle |  |
|  |  | Poorer |  |
|  |  | Poorest |  |
|  | Distance (in km) | | -0.025 (0.006)*** |
| **Homophily Variables** | Age difference between CHW & female | | -0.001 (0.001) |
|  | Education difference between CHW & female | | -0.020 (0.008)* |
|  | Wealth difference between CHW & female | | -0.0002 (0.005) |
|  | Ethnic group difference between CHW & female | | -0.010 (0.015) |
| Village Dummy Variable | Village 2 | | -0.142 (0.009)*** |
|  | Village 3 | | -0.223 (0.009)*** |
|  | Village 4 | | -0.309 (0.228)*** |
|  | Village 5 | | -0.035 (0.016)* |
|  | Village 6 | | -0.316 (0.014)*** |
|  | Village 7 | | -0.298 (0.019)*** |
|  | Village 8 | | -0.299 (0.006)*** |
|  | Village 9 | | -0.174 (0.012)*** |
|  | Village 10 | | -0.140 (0.012)*** |
|  | Village 11 | | -0.0260 (0.018)*** |
|  | Village 12 | | -0.061 (0.009)*** |
|  | Village 13 | | 0.296 (0.012)*** |
|  | Village 14 | | -0.050 (0.017)** |
|  | Village 15 | | -0.020 (0.013) |
|  | Village 16 | | -0.162 (0.013)*** |
|  | Village 17 | | -0.060 (0.009)*** |
|  | Village 18 | | -0.225 (0.010)*** |
|  | Village 19 | | -0.031 (0.013)* |
|  | Village 20 | | -0.148 (0.013)*** |
|  | Village 21 | | -0.303 (0.010)*** |
|  | Village 22 | | -0.320 (0.015)*** |
|  | Village 23 | | -0.144 (0.013)*** |
|  | Village 24 | | -0.349 (0.010)*** |
|  | Village 25 | | -0.152 (0.015)*** |
|  | Village 26 | | -0.292 (0.014)*** |
|  | Village 27 | | -0.299 (0.014)*** |
|  | Village 28 | | -0.309 (0.016)*** |
|  | Village 29 | | -0.223 (0.041)*** |
|  | Village 30 | | -0.328 (0.009)*** |
|  | Village 31 | | -0.173 (0.011)*** |
|  | Village 32 | | -0.242 (0.013)*** |
|  | Village 33 | | -0.257 (0.014)*** |
|  | Village 34 | | -0.178 (0.019)*** |
|  | Village 35 | | -0.287 (0.013)*** |

^*^p < 0.05, ^**^ p < 0.01, ^***^ p < 0.001
